# Behavioral reconsolidation interference with episodic memory within-subjects is elusive

**DOI:** 10.1016/j.nlm.2018.03.004

**Published:** 2018-04

**Authors:** Daniel A. Levy, Rotem Mika, Cecilia Radzyminski, Shir Ben-Zvi, Roni Tibon

**Affiliations:** a Baruch Ivcher School of Psychology, The Interdisciplinary Center, Herzliya, Israel; b Department of Psychology, Tel-Aviv University, Tel Aviv, Israel; c Sackler School of Medicine, Tel-Aviv University, Tel Aviv, Israel; d MRC Cognition and Brain Sciences Unit, University of Cambridge, UK

**Keywords:** Reconsolidation, Memory, Episodic, Interference, Recognition

## Abstract

In studies of behavioral reconsolidation interference, reactivation of a consolidated memory using some form of reminder is followed by the presentation of new information that can cause interference with that memory. Under these conditions, the interference not only impairs retrieval by indirect processes such as cue interference, but supposedly disrupts the original memory trace directly. Almost all studies of behavioral reconsolidation interference in episodic memory in humans have employed between-subjects paradigms, and deduced reminder effects from intrusion errors. Such studies might introduce confounds arising, for example, from differences in retrieval strategies engendered by the pre-test treatments. We therefore set out to examine whether behavioral reconsolidation interference in episodic memory might be demonstrated within-subjects and by direct memory strength rather than intrusion errors. In three separate experiments, we attempted to disrupt reconsolidation of episodic object-picture memory using a reminder + retroactive interference manipulation. We applied the manipulation over three consecutive days, using a forced-choice recognition test without intrusions from interfering learning, keeping all other study and test parameters constant. No effects of reminder-potentiated interference were observed for measures of accuracy, response times, subjective expressions of recollection, or levels of confidence, as substantiated by Bayesian analyses. These results highlight the difficulty of observing clear behavioral reconsolidation interference effects within-subjects in human episodic memory, and provide some indications of what might be boundary conditions for its demonstration.

## Highlights

• We assessed behavioral reconsolidation interference in episodic memory.
• Employed within-subjects paradigm measuring direct memory strength.
• Three experiments found no effects of reminder-potentiated interference.
• Null effects were substantiated by Bayesian analyses.
• Highlights boundary conditions for behavioral reconsolidation interference effects.

## 1 Introduction

One of the most interesting topics of inquiry in contemporary memory research is the phenomenon of reconsolidation. Converging evidence from a variety of experimental paradigms in several animal species, and more recently in humans, indicates that when consolidated memory traces are activated, they become labile, and subject to modification or erasure if reconsolidation processes are disrupted (Dudai, 2012, Lee et al., 2017). A promising aspect of this phenomenon is its therapeutic potential – the prospect of modifying or even erasing aversive or maladaptive memories. However, most demonstrations of memory modification through reconsolidation interference have relied on pharmacological agents, and their use in humans is not trivial. Therefore, there have been several attempts to disrupt reconsolidation of human memory by behavioral interventions (reviewed by Agren, 2014, Hupbach et al., 2015, Schiller and Phelps, 2011, Scully et al., 2017).

In studies of behavioral reconsolidation interference, reactivation of a consolidated memory using some form of reminder is followed by the presentation of new information that can cause interference with that memory. Under these conditions, the interference is supposed not only to impair retrieval by indirect processes such as cue interference, but to directly disrupt the original memory trace. Reconsolidation interference is asserted to have occurred if retrieval of target memory information is more greatly impaired when interference is preceded by a reminder compared to a condition in which reminder reactivation was not followed by new interfering information, or a condition in which the new information was not preceded by a reminder (Hupbach et al., 2015, Scully et al., 2017).

Several studies have investigated whether such behavioral reconsolidation interference may degrade episodic memories (Scully et al., 2017). Such a process might be especially important for therapeutic interventions to ameliorate intrusive traumatic recollections. For example, it was recently demonstrated that playing the computer game Tetris after reminder activation of memories of a traumatic film reduced subsequent intrusive memories of that film (James et al., 2015). More prosaically, reconsolidation interference in episodic memory might take the form of source memory deterioration, exemplified by list intrusions during recall. For example, one early study indicated that learning a new list of objects following the reactivation of a previously learned list caused items from the second learning to be mistakenly recalled as having appeared in the first list (Hupbach, Gomez, Hardt, & Nadel, 2007).

Almost all studies of behavioral reconsolidation interference in episodic memory in humans have employed between-subjects paradigms, comparing effects of treatment groups. While providing valuable information about the scope of reconsolidation effects, such studies do not readily enable examination of the physiological bases of the effects in humans (e.g., by using non-invasive neuroimaging techniques). Perhaps more fundamentally, if reconsolidation interference effects are only seen in between-subjects paradigms, it becomes difficult to rule out possible additional factors that might be responsible for effects on retrieval, such as strategic changes in retrieval orientation engendered by preceding reminders. Such potential confounds make it difficult to determine whether the intervention resulted in modification of the mnemonic representations themselves, as reconsolidation interference is asserted to cause.

To the best of our knowledge, within-subject examination of episodic memory reconsolidation interference has rarely been attempted. In one study (Zhu et al., 2016), incorporating a series of experiments, participants engaged in pair associate learning in which two cues elicited each response target. In the interference phase, only one of those cues was manipulated by being associated with a set of additional responses, either following a strong reminder of the original pairing or without a reminder. Memory for the targets was subsequently probed with both the manipulated cue and with the non-manipulated cue. Interestingly, the reminder presentation increased the deleterious effect of interference only on recall elicited by the non-manipulated cue, except when the interference was conducted immediately after the learning phase. Additionally, in this design, two different cues are linked to the same response, thus reducing association strength (i.e., a blocking effect). Thus, reconsolidation interference in the strict sense was not observed.

A within-subjects manipulation of selective reminder reactivation formed part of the design in the study of Hupbach and Dorskind (2014), along with a between-subjects manipulation, in which some participants underwent cold-pressor stress intended to interfere with reconsolidation. The researchers report that the reminder procedure enhanced later recall in the control group, but did not affect the stress group. Thus, while this study demonstrated that selective reminder reactivation may interact with subsequent stressors, the stressor might have prevented the memory-enhancing effects of reactivation rather than specifically impairing reconsolidation of the reactivated representations. Additionally, it remains to be determined whether such effects would be observed in a purely within-subjects paradigm. Similarly, Forcato et al. (2016) also employed a mixed within-subjects (manipulation of reminder type) and between-subjects (manipulation of interference) design. They report better subsequent cued recall in the reactivation group than in the reactivation-interference group in the syllable-reminder condition but not in the pair-reminder and picture-reminder conditions. Here too, the effects of the type of reminder were observed in the context of a between-subjects paradigm. It is therefore important to assess potential reconsolidation interference effects in a more basic within-subjects design.

We therefore set out to examine whether behavioral reconsolidation interference in episodic memory might be demonstrated when both reminder and interference conditions were manipulated within-subjects. We employed a visual object recognition memory task, in which participants studied a series of object pictures on Day 1, underwent a selective reminder/interference manipulation on Day 2, and took a two-alternative forced choice recognition test requiring discriminating the originally studied pictures from novel lures (other exemplars of the same objects), on Day 3. We followed the basic approach of comparing the effects of post-consolidation interference between a condition in which target memories were activated immediately before the interference and a condition in which there was no such activation, and additionally compared conditions of encoding-alone and encoding with reminder. The question was whether reminder-potentiated specific retroactive interference would most greatly impair subsequent recognition memory compared with the other conditions. As documented below, however, despite several attempts to titrate reminder strength, we were unable to find evidence that this approach led to reconsolidation interference as expressed in poorer recognition accuracy, slower response times, or subjective reports of recollective strength or confidence.

## 2 Experiment 1

### 2.1 Methods

#### 2.1.1 Participants

Participants were 40 healthy young adults (10 males; mean age 22.9 years, SD = 1.8 years, range 20–27), with normal or adjusted-to-normal vision. All were undergraduate students who volunteered in return for academic requirement credit or payment. Informed consent was obtained from all participants for a protocol approved by the Interdisciplinary Center's Institutional Review Board. Five other participants were excluded from the analyses: two who did not properly follow instructions, two whose overall performance was more than 2 SD weaker than group mean recognition success and close to chance, and one due to data corruption after the experiment.

#### 2.1.2 Stimulus material

**Fig. 1:**
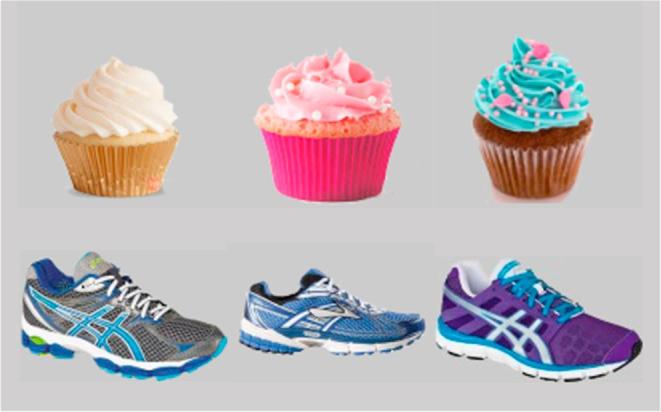
Examples of object picture exemplars employed in all three experiments.

Pictures of 200 simple common objects (e.g., apple, sunglasses, dog) collected from various internet sites were used as stimuli. Three versions of each object type (examples in Fig. 1) were edited to be comparable in size, detail, and resolution. The three versions of the same objects were randomly assigned to three lists (A, B, or C), with assignment of lists to roles as recognition targets studied on Day 1 and tested on Day 3, interfering study condition on Day 2, or recognition test lures on Day 3 counterbalanced across participants.

#### 2.1.3 Procedure

The experiment was conducted using E-Prime 2 (Psychology Software Tools). The experimental procedures were carried out on three consecutive days. On the first day, after providing informed consent, participants were serially shown a set of 200 objects onscreen (from one of the lists A, B or C). They were instructed to memorize the visual details of these objects for a memory test that would be administered at an unspecified later time. To facilitate memory formation via deep encoding, participants made self-paced liking judgements for each object on a scale of 1–5, using corresponding buttons on the keyboard, with 1 indicating “dislike very much” and 5 indicating “like very much”. Each keypress triggered presentation of the next stimulus following a delay of 500 ms. A rest break was provided after each 50 pictures. Participants were instructed to return to the lab at the same time the following day.

On the second day of the experiment, participants initially performed a reminder activity. They were shown the names of 100 of the objects they saw the previous day, and asked to report how well they remember the actual picture of that object from the day before, using three marked buttons on the keyboard, representing “clearly”/“hardly”/“not at all”. Thereafter, participants were shown 100 alternative object pictures drawn from a second picture set (e.g., participants who saw list A on Day 1, saw pictures drawn from either lists B or C). This series consisted of 50 objects for which reminders had been previously provided in the first part of Day 2 (forming the Reminder + Interference condition), and another 50 which were not (forming the Interference Only condition). Participants were instructed to memorize this second set as well, for a memory test that would be held on the following day (which was not carried out in practice).

Pilot testing had indicated that interference in the critical recognition task of Day 3 could be anticipated only if strong interference was generated on Day 2. That result is in consonance with the report of Wichert, Wolf, and Schwabe (2013) that multiple interference exposures of new pictures after reactivation of prior learning is necessary for reconsolidation interference effects to emerge. Therefore, for this second set of 100 pictures, participants were asked to perform three tasks with each object picture: liking, symmetry and color judgments, executed in a fixed order. Since liking judgments were also performed on Day 1, participants were told not to try to be consistent with their prior liking ratings. In the second task, color judgment, participants were asked to indicate, as rapidly and accurately as possible, how many colors they saw in the picture displayed, using the following buttons specially labeled on the keyboard; “1–3”/“3–4”/“5+”. In the third task, symmetry judgement, participants were asked to judge whether each object is symmetrical (using the “V” key) or non-symmetrical (using the “X” key). The object picture was re-displayed before each judgment. In all tasks, each keypress triggered presentation of the next stimulus following a delay of 500 ms. Rest breaks were provided between tasks.

Thus, through the procedures of Day 2, four experimental conditions were created: (1) 50 objects that were only encoded in Day 1 and not referenced at all on Day 2 (Encoding-only); (2) 50 objects that were encoded on Day 1 and only reminded on Day 2 (Reminder); (3) 50 objects that were encoded on Day 1, and of which an alternative version was studied in the second part of Day 2, intended to create interference in the subsequent recognition test (Interference); (4) 50 objects encoded on Day 1, and reminded and subsequently interfered with on Day 2 (Reminder + Interference). Upon completion of the second learning activity, participants were instructed to return to the lab at the same time the following day.

On the third day of the experiment, participants performed a two-alternative forced choice recognition task, in which they were to discriminate between pictures studied on Day 1 and novel alternative exemplars of the same object, drawn from the third set of pictures that had not previously been seen. The two alternatives were displayed horizontally onscreen, with old and new exemplars randomly placed on either the left or the right side of the screen. Importantly, discriminating the targets from the lures requires detailed memory of the originally studied items, and cannot rely on gist memory alone. Participants were asked to use left and right arrows to choose which object was the one they saw on Day 1. Accuracy and response time measures were collected. We hypothesized that even if potentiation of interference by reminders did not affect global recognition accuracy, it might diminish the sense of episodic recollection of the target stimulus. Therefore, after choosing the stimulus they believed that they had studied on the first day, participants were asked to indicate whether they vividly remembered the chosen exemplar with its contextual details (“Remember” response, indicted by a key marked with the letter R), or just knew that it was the exemplar that had been studied (“Know” response, indicated by a key marked with the letter K). That keypress triggered presentation of the next stimulus, following a delay of 500 ms. A rest break was provided after each 50 trials.

Since object names were used as Day 2 reminders, names that did not signify the objects for the participant would not be an appropriate reminder. Therefore, following completion of all memory tests, participants were shown pictures from the set they had seen on the first day and asked to record their names. Items for which names were given that were substantively different than the names used for reminders on Day 2 were removed from subsequent analyses for that participant. On average, 1.7 items per participant were thus removed, with a maximum of 11 items removed in one participant. Trials in which response times (RTs) were three standard deviations or more longer than the individual participant's other RTs were removed from RT analyses.

Measures of accuracy, RTs, and the proportion of 'Remember' compared to 'Know' responses following correct recognition were submitted to analyses of variance to examine main effects of reminder and interference conditions – and crucially, their interaction. These analyses were accompanied by with planned comparisons testing the critical prediction that reminders would potentiate reconsolidation interference, such that memory in the Reminder + Interference condition should be weaker than in the Interference condition. As noted below, observed null effects were examined with Bayesian analyses. All statistical analyses were performed using R version 3.4.1 and RStudio Version 1.0153, with packages BayesFactor (Morey & Rouder, 2015), ez (Lawrence, 2016), and psych (Revelle, 2017).

### 2.2 Results

**Fig. 2:**
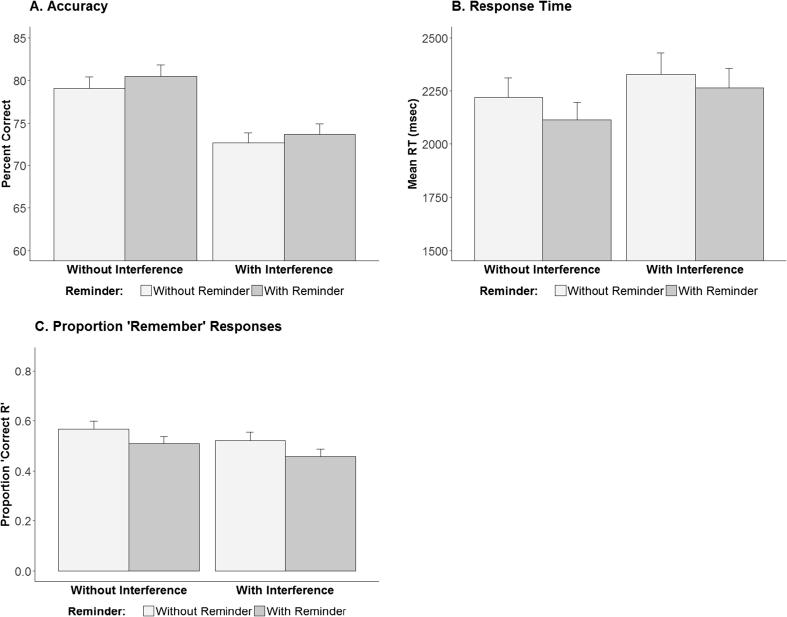
Accuracy (Panel A), response time (Panel B), and the proportion of 'Remember' compared to 'Know' responses following correct recognition judgments (Panel C) measures, for the two-alternative forced choice picture recognition test in Experiment 1. Error bars indicate SEM.

We examined the effects of reminder, interference and the interaction between them on accuracy and response times at test, by using a 2 × 2 repeated measures ANOVA, with Interference (with/without) and Reminder (with/without) as repeated factors. The results of these analyses are portrayed in Fig. 2, Panels A and B.

For accuracy, the only significant effect was a strong deleterious effect of Interference, *F*(1, 39) = 40.81, *p* < 0.001, *Ƞ^2^G* = 0.14. Importantly, our prediction of greater accuracy rates in the Interference condition relative to the Interference + Reminder condition was not confirmed, *t*(39) = −0.77, *p* = 0.78; numerically, the former condition actually yielded slightly *better* memory. For response times, the analysis revealed a significant main effect of Interference, *F*(1, 39) = 8.54, *p* < .01, *Ƞ^2^G* = 0.013, and a significant main effect of Reminder, *F*(1, 39) = 6.77, *p* < 0.05, *Ƞ^2^G* = 0.01, with no interaction between these factors. Our prediction of reduced response times in the Interference condition relative to the Interference + Reminder condition was not confirmed, *t*(39) = −1.32, *p* = 0.9; rather, a trend in the opposite direction emerged.

We then analyzed the effects of reminder and interference manipulations on the relative proportions of reported 'Remember' responses, representing more subjective aspects of memory. Due to file corruption, these data were only available for 35 of the 40 participants. A 2 × 2 repeated measures ANOVA was used, with percent of correct 'Remember' responses out of all correct responses (i.e., correct Remember/(correct Remember + correct Know) as the dependent measure (Fig. 2, Panel C). The analysis revealed a significant main effect of Interference, *F*(1, 34) = 4.91, *p* < .05, *Ƞ^2^G* = 0.02, and a significant main effect of Reminder, *F*(1, 34) = 11.8, *p* < 0.01, *Ƞ^2^G* = 0.03, showing (somewhat surprisingly) a significant lower proportion of correct Remember responses following reminders. As predicted, our planned comparison yielded a lower percentage of correct Remember responses in the Reminder + Interference compared to the Interference condition, *t*(34) = 2.61, *p* < 0.01. However, since for this measure, the reminders also yielded a main effect of decreasing Remember responding (that is, a similar decrease was observed for the Reminder condition relative to the Encoding-only condition), the lower proportion of Remember responding in the Reminder + Interference condition relative to the Interference condition is likely due to inclusion of a reminder in the former, and does not provide evidence that the reminders had an interactive effect with interference.

Our main prediction, of weaker, slower, or less richly recollective memory following interference preceded by a reminder than following interference alone was generally not borne out by these analyses. As with any form of classical null-hypothesis testing, however, absence of evidence is not evidence of absence. We therefore adopted recent proposals to use Bayesian factors to compare null and alternate hypotheses. We used a one-sided Bayesian *t*-test with a Cauchy prior scaled at sqrt(2)/2 (medium scaling), to compare two hypotheses: that the accuracy rates in the Interference condition do no differ from accuracy rates in the Interference + Reminder condition (that is, that standardized effect size is 0; the so-called “null hypothesis”), or that accuracy rates in the Interference condition are higher than in the Interference + Reminder condition (that is, that the standardized effect size is bigger than 0). This analysis supported the null hypothesis, which was preferred by a Bayes factor of 9.71. We ran the same analysis for reaction times, this time contrasting the null hypothesis with the alternate hypothesis of reduced reaction times in the Interference condition relative to the Interference + Reminder condition. For this analysis, the null hypothesis was preferred by a Bayes factor of 12.63. The data thus provide strong evidence against the hypothesis of reduced memory following interference that is preceded by a reminder. Finally, we ran this analysis for the proportion of correct Remember responses, which yielded moderate evidence in support of the alternate hypothesis (BF = 6.6). As previously noted, however, given that this measure shows a general decrease in the proportion of correct Remember responses following a reminder even without interference, this result does not provide evidence that the reminders had an interactive effect with interference.

### 2.3 Discussion

In our first examination of the potential effects of reminder-potentiated interference, we failed to find such interactive effects on either objective (accuracy and response times) or subjective (reported strength of recollection). The predicted interaction between Reminder and Interference manipulations was not observed despite the fact that both manipulations individually did significantly influence either accuracy or response time measures.

An unexpected finding in Experiment 1 was that the Encoding-only condition was characterized by a higher percentage of correct 'Remember' responses than the (marginally more accurate) Reminder condition. It is not clear why this effect was obtained. We speculate that perhaps the reminder activation of the ‘gist’ memory of the item type in the absence of re-viewing of the particular stimulus led to a decline in subjective re-experiencing at retrieval, even while improving the objective memory for that stimulus.

Our failure to observe the expected interactive effect of reminder on interference led us to wonder it was absent because the reminders were all presented in a single list, while the interfering stimuli were presented in a later phase of the session. That might have resulted in some fashion in the resetting of reminder-based activation before the interference affected the memory traces. In Experiment 2, we therefore went on to employ a slightly different experimental paradigm, in which the reminders and the interfering stimuli are presented in close proximity, interleaved in a single series of alternating judgments. If proximity of reminder and interference is crucial for interactive effects to emerge, this paradigm should yield stronger evidence of the interaction.

## 3 Experiment 2

### 3.1 Methods

#### 3.1.1 Participants

Participants were 40 healthy young adults (10 males; mean age 22.1 years, SD = 3.0 years, range 19–31), with normal or adjusted-to-normal vision. All were undergraduate students who volunteered in return for academic requirement credit or payment. Informed consent was obtained from all participants for a protocol approved by the Interdisciplinary Center's Institutional Review Board. One other participant was excluded from the analyses due to failure to properly follow instructions, as indicated by random performance.

#### 3.1.2 Materials and procedure

Materials, Day 1 study procedures, and Day 3 test procedures were identical to Experiment 1. On Day 2 of the experiment, participants were reminded of 100 objects and exposed to interfering exemplar pictures of 100 objects, creating the same four conditions as in Experiment 1. However, in Experiment 2, the two activities of Day 2 were interleaved. Each trial unit began with a reminder phase, in which participants were shown an object name and asked to report how well they remember the actual picture of that object from the previous day, using 3 buttons on the keyboard; “clearly”/“hardly”/“not at all”. In contrast to Experiment 1, however, immediately after each such report, they were shown an object from a different list (an Interference object; i.e., if participants saw an object from list A on Day 1, they saw either an object from list B or C), which they were again asked to memorize for a memory test. For half of the trials, the interference object was an alternate exemplar of the same object that was just reminded (becoming a case of the Reminder + Interference condition), and for the other half, the interference was an alternate exemplar of a different object than the one just reminded (such that the reminded stimulus became a case of Reminder condition, and the alternate exemplar became a case of Interference condition). As in Experiment 1, participants were asked to perform three tasks for each interference object; liking, symmetry and color judgment, as described above. The next trial unit began with the next name-cued memory assessment as a reminder. This procedure yielded the same four conditions as Experiment 1, the only difference being that the Reminder, Interference, and Reminder + Interference conditions were all created in the same process on Day 2; the Encoding-only condition stimuli were, as in Experiment 1, only seen on Day 1.

Day 3 accuracy and response time measures were collected as in Experiment 1. As in Experiment 1, following completion of all memory tests on Day 3, participants were shown pictures from the set of that they had seen on the first day and asked to record their names. Items for which names were given that were substantively different than the names used for reminders on Day 2 were removed from subsequent analyses for that participant. On average, 1.85 items per participant were thus removed, with a maximum of 10 items removed in one participant. Trials in which response times (RTs) were three standard deviations or more longer than the individual participant's other RTs were removed from RT analyses.

### 3.2 Results

**Fig. 3:**
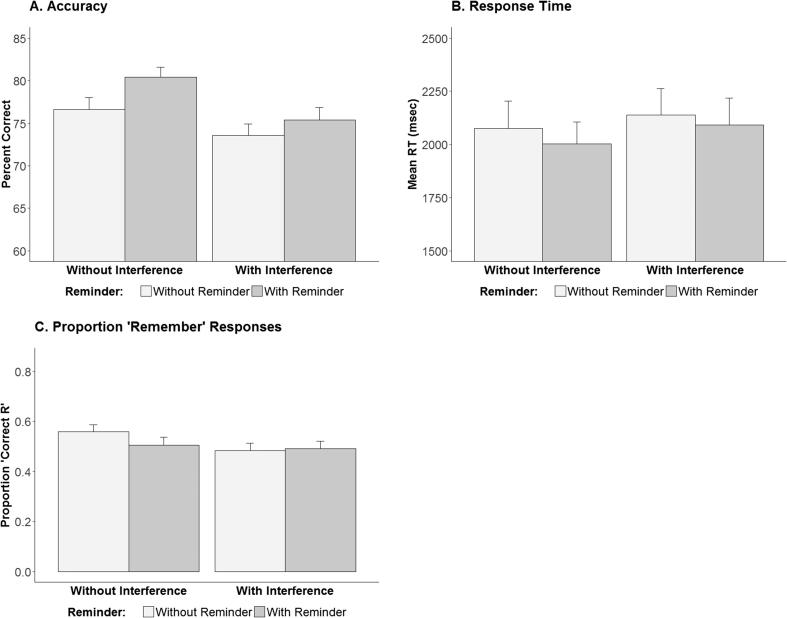
Accuracy (Panel A), response time (Panel B), and the proportion of 'Remember' compared to 'Know' responses following correct recognition judgments (Panel C) measures for two-alternative forced choice picture recognition test in Experiment 2. Error bars indicate SEM.

As in the prior experiment, we examined the effects of reminder, interference and the interaction between them in repeated measures ANOVAs for accuracy, response times, and proportion of Remember responses following correct recognition judgments, as portrayed in Fig. 3. For accuracy, this revealed a main effect of interference, *F*(1, 39) = 12.96, *p* < 0.001, *Ƞ^2^G* = 0.05, with reduced accuracy rates following interference, a main effect of reminder, *F*(1, 39) = 16.63, *p* < 0.001, *Ƞ^2^G* = 0.03, with increased accuracy rates following reminders, and no interaction between the two factors. Critically, our planned comparison between Interference and Interference + Reminder did not support our prediction, *t*(39) = −1.57, *p* = 0.94. For response times, the analysis only revealed a main effect of interference, *F*(1, 39) = 9.24, *p* < 0.005, *Ƞ^2^G* = 0.003, and no support for our prediction, *t*(39) = 1.56, *p* = 0.94. Finally, for the proportion of correct Remember responses, the analysis revealed a significant main effect of interference, *F*(1, 39) = 9.56, *p* < 0.005, *Ƞ^2^G* = 0.015, as well as a significant interaction between the two factors, *F*(1, 39) = 5.88, *p* < 0.05, *Ƞ^2^G* = 0.01. Nevertheless, decomposition of the interaction revealed that it is driven by an unexpected *increase* in proportion of Remember responses in the Encoding-only condition compared to the Reminder condition, *t*(39) = 2.99, *p* < 0.01, but only when there was no interference. In other words, the predicted difference between Interference vs. Interference + Reminder was not observed, *t*(39) = −0.42, *p* = 0.66.

Our main prediction, of poorer memory following interference preceded by a reminder than following interference alone, was thus not borne out by these ANOVAs in this experiment either. As in Experiment 1, we further scrutinized these patterns of results by examining Bayesian factors to compare null and alternate hypotheses. These analyses revealed strong support for the null hypotheses of no difference between the Reminder condition and the Reminder + Interference condition, for both accuracy (BF = 13.97) and reaction times (BF = 13.9), and moderate support for the null hypothesis regarding the proportion of Remember responses (BF = 7.86).

### 3.3 Discussion

In this experiment, we observed tangible effects on remembering of both reminder and interference manipulations alone, the former strengthening memory and the latter weakening it, but did not observe potentiation of interference by reminders. This was obtained under conditions of interleaved processing of reminders and interference. Thus, the absence of the effect that we observed in Experiment 1 cannot be attributed to insufficient interaction between the reminder and interference stages.

It is interesting to note that unlike Experiment 1, here memory for the Reminder condition was significantly stronger than the Encoding-only condition. It is possible that such an effect emerged because in this paradigm the reminders are given just before the interfering stimuli. This may cause the reminders to become differentially more efficient in strengthening memory for the original items when they are not followed by specific interference by a competing version of the same stimulus, possibly due to more elaborative (re)encoding of the original stimulus.

We were left with the question of why we did not observe the predicted potentiation of interference by reminder activation of target representations. One reason for the absence of such effects might be that the reminder employed, directly referencing the previously encoded stimuli, was so strong that it overshadowed the reminder modulation of interference. Indeed, several studies have put forth the claim that only weak reminders will lead to reconsolidation interference (e.g., Hupbach, 2011), although such claims are not supported by a recent meta-analysis of reconsolidation studies (Scully et al., 2017). To examine this possibility, in Experiment 3 we used a very weak reminder, i.e., one that made no explicit reference to the encoding episode. Rather, participants were presented with pairs of object names, and asked to decide which was more expensive. This deep encoding task requires making reference to the semantic information related to the object name, which by spreading activation might minimally activate representations of the studied object picture without bringing it to consciousness. This manipulation was only applied to half of the stimulus exemplars, providing a parallel structure with the four conditions of the two prior experiments. If weak reminders can potentiate interference, then this manipulation might be expected to yield such effects.

## 4 Experiment 3

### 4.1 Methods

#### 4.1.1 Participants

Participants were 40 healthy young adults (5 males; mean age 20.9 years, SD = 2.6 years, range 19–31), with normal or adjusted-to-normal vision. All were undergraduate students who volunteered in return for academic requirement credit or payment. Informed consent was obtained from all participants for a protocol approved by the Interdisciplinary Center's Institutional Review Board.

#### 4.1.2 Procedure

Day 1 of the experiment was executed with the same protocol as in the two prior experiments. On Day 2, participants performed two tasks. In the first task, they were shown the names of 100 of the items that they had studied on Day 1, in horizontal pairs, midscreen and spaced for comfortable reading, in a black 18-point font on a white background. They were asked to indicate which item they thought is more expensive, by pressing either the right or left of two designated keys on a standard keyboard (marked with stickers as 'R' or 'L'). The selection of the 100 item names presented for this pricing judgment (out of 200 items studied on the previous day), and the relative location of each item onscreen, was counterbalanced across participants. The second task of Day 2 was an interference manipulation, which was conducted as in Experiment 1.

On Day 3, participants performed a two-alternative forced choice recognition tasks, initially choosing the exemplar studied on Day 1 as in Experiments 1 and 2. However, in this experiment, we employed a more continuous measure of memory strength than the Remember/Know judgments used previously. Here, in each trial participants were asked to provide a rating of their confidence in the recognition judgment they had made, using a 5-point scale (with a guide indicating: 5 = absolutely sure, 4 = fairly sure, 3 = feeling of knowing without strong confidence, 2 = very weak memory, 1 = totally guessing). We made this change with the rationale that perhaps providing a continuous scale of subjective remembering might reveal effects that were hidden by the binary character of the Remember/Know responses. Accuracy, response time, and confidence measures were collected, and analyzed using the same methods as in the prior experiments.

### 4.2 Results

**Fig. 4:**
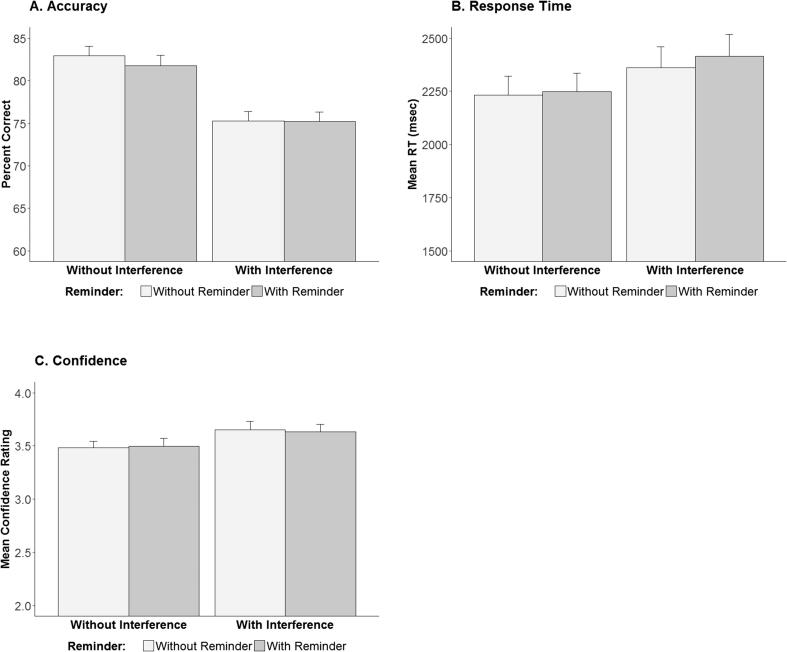
Accuracy (Panel A), response time (Panel B), and confidence (Panel C) measures for two-alternative forced choice picture recognition test in Experiment 3. Error bars indicate SEM.

As in the prior experiments, we examined the effects of reminder, interference and the interaction between them in repeated measures ANOVAs for the dependent measures of accuracy and response times. We ran an additional ANOVA, with the same independent variables, and with mean confidence rating as the dependent measure. For all of these measures (Fig. 4), the analysis revealed a significant main effect of interference, [for accuracy: *F*(1, 39) = 67.35, *p* < 0.001, *Ƞ^2^G* = 0.2; for response times: *F*(1, 39) = 18.61, *p* < 0.001, *Ƞ^2^G* = 0.02; for confidence: *F*(1, 39) = 4.93, *p* < 0.05, *Ƞ^2^G* = 0.03], but no other significant effects. In addition, our planned comparison revealed no advantage for the Interference condition over the Interference + Reminder condition in any of these measures [for accuracy: *t*(39) = 0.05, *p* = 0.48; for response times: *t*(39) = 1.47, *p* = 0.08; for confidence: *t*(39) = 0.37, *p* = 0.35].

Finally, in this experiment Bayesian analyses provided moderate evidence in favor of the null hypothesis for accuracy rates (BF = 5.66) and for confidence (BF = 4.28) and anecdotal evidence in favor of the null hypothesis for reaction times (BF = 1.19). Importantly, however, the prediction of worse memory following interference preceded by a reminder than following interference alone was not confirmed in this experiment either.

## 5 General discussion

In three separate experiments, we attempted to disrupt reconsolidation of episodic object-picture memory using a reminder + retroactive interference manipulation. We applied the manipulation in a within-subjects paradigm, using a forced-choice recognition test without an intrusion component (i.e., no stimuli from Day 2 were presented in the memory tests to cause interference-to-target confusion), keeping all other study and test parameters constant. No effects of reminder-potentiated interference were observed for measures of accuracy, response times, subjective expressions of recollection, or levels of confidence, in any of these experiments, as substantiated by Bayesian analyses. Seemingly, the alternative exemplars presented on Day 2 engendered classic cue-overload retroactive interference that caused difficulties in discrimination between originally studied exemplars and novel foils on Day 3, but may not have disrupted the stored representations themselves.

A key motivation of the present study was to determine whether reminder-potentiated reconsolidation interference in episodic memory could be demonstrated within-subjects. Consonant with the report of Zhu et al. (2016), we were not successful in eliciting that effect. The question remains why such effects were not obtained in the present research despite being reported in other studies. We will consider several possible factors that might account for this.

The first possible factor is the strength of the reminder used to cause pre-interference reactivation. The primary difference between the three experimental paradigms herein reported was the strength of the reactivation of the consolidated target memory before interference. Hupbach et al. (2007) have asserted that strong, direct reactivations should induce less memory change than indirect reactivations, as strong reactivation might strengthen the original representation through rehearsal and vitiate the effects of interference. However, a meta-analysis of reconsolidation effects by Scully et al. (2017) did not confirm that assertion, with effect sizes not found to be modulated by reactivation methods. Accordingly, any of the methods we employed might in principle have led to reconsolidation interference – though in practice they did not.

Similarly, it might be argued that the object names employed as reminders do not actually trigger reconsolidation at all, and that the original memories are not made labile by those reminders. This seems a less likely possibility in Exps. 1 and 2 in which the names were cues for the assessment of memory strength of the individual items, but might be relevant to Exp. 3 in which the names of the objects were presented in the context of a cognitive judgment unrelated to the encoding episode. However, given that in other studies purporting to observe reconsolidation interference, the triggering of labilization was attributed to reactivation engendered by a general reminder of the encoding context, the repeated semantic processing of the name of a studied object employed in that experiment seems more likely to yield reactivation of the studied item.

Another possibility is that since the recognition test we employed can be performed using familiarity without need for recollection (Yonelinas, 2002), the absence of a reconsolidation interference effect might be because the memory test was not sufficiently challenging. However, since in this study lures were the same item types as the studied targets, detailed memory and not gist familiarity was required for correct recognition. We chose the three-exemplar target-interference-foil paradigm precisely because in this paradigm specific retroactive interference to the representation of the studied target makes it difficult for participants to distinguish it from a novel foil of the same object type. In other words, the similarity between targets, interferers, and foils makes it more challenging to retrieve the specific structural/perceptual features of the target. Indeed, performance was far from ceiling, and many participants found the task quite challenging. Importantly, the meta-analysis of Scully et al. (2017) indicated that memory test type (recall or recognition) did not affect the degree of the reactivation-induced memory change. Therefore, it might have been expected that we find the effect on recognition, but this was not observed. Furthermore, the results show that there was no effect on the proportions of correct ’Remember’ responses, which indicates that the effect is not absent just because participants are making familiarity-based judgments.

Another possible reason that we did not find evidence for reconsolidation interference is that the reminder-interference manipulation was conducted only 24 h after the original learning. While the intervening night sleep should afford some degree of consolidation, a meta-analysis of reconsolidation effects by Scully et al. (2017) indicated that reactivation before interference only moderately affected memories that were between 24 and 48 h old, yielding more significant changes in memories greater than one week old. For example, among the cases of behavioral reconsolidation interference in episodic memory, Schwabe and Wolf (2009; using a recognition test for emotional and neutral pictures) and Wirkner, Löw, Hamm, and Weymar (2015; using story recall) observed the effects of reactivation on interference when memory was tested one week after encoding. Wichert, Wolf, and Schwabe (2011) found evidence for retrieval-potentiated interference with recall of neutral and emotional pictures, when the interference was administered 7 days after, but not 1 day or 28 days after encoding. Summarizing findings from a variety of reconsolidation studies (including those that used pharmacological interventions), Scully et al. (2017) propose that very recent memories are not sufficiently consolidated, such that subsequent manipulations might equally affect both reactivated and non-reactivated memories. The possibility of finding reconsolidation interference using the present methods with much longer consolidation intervals might be examined in future studies.

Finally, it could be argued that the form of interference we employed, involving competing exemplars of the studied stimuli, itself served as a reminder, such that any observed effects of interference are necessarily effects of reconsolidation interference. We believe that this aspect of the procedures cannot fully account for the reported results, for several reasons. Firstly, while in Experiment 1 the reminder activity and the interference activity were blocked (such that participants first performed the reminder activity for all objects, and then the interference activity), in Experiment 2 these two activities were interleaved. Thus in Experiment 2, participants viewed an object name triggering recall of the original image, and then immediately viewed a different image. In this procedure, there is an immediate mismatch between the two instances of the object (the retrieved and the viewed), and so the design of Experiment 2 seems to induce increased prediction error (PE) relative to the design of Experiment 1. It has been asserted that PE is mandatory during reactivation session in order to initiate reconsolidation, and that memory reconsolidation depends on the amount of PE (reviewed by Fernández, Boccia, & Pedreira, 2016). Therefore, we would expect to observe reminder-potentiated reconsolidation interference effects at least in Exp. 2, but that was not apparent. Secondly, we have grounds to expect that the pre-interference reminder would engender differential effects above and beyond the reminder strength of the interference stimuli. In the application of reconsolidation interference to extinction of fear conditioning (e.g., Schiller et al., 2010, Agren et al., 2012), the extinction training certainly serves as a reminder of the conditioning no less than the alternative exemplars presented in the interference stage of the current study. Yet, in those studies, the presentation of a reminder cue a few minutes before the beginning of extinction training has a strong effect on the efficacy of the extinction. The extinction of conditioning is indeed a different type of memory process than episodic interference. However, to the extent that, as a general principle, reminder-based activation of previously acquired traces leaves such representations more vulnerable to interference, we should have seen differentially weaker subsequent memory in the reminder condition in the current paradigm – but did not. Thus, while the possibility that the interfering images also served as reminders cannot be completely ruled out, we were not able to engender any interaction between prior reminders and that interference procedure.

It should be noted that in several of the prior studies reporting reminder-potentiated reconsolidation interference, such effects were expressed not in forgetting of studied materials, but in intrusion effects – essentially source memory failures, in which items from distractor lists are reported as belonging to target lists (Hupbach et al., 2007, [Bibr b0070][Bibr b0065], Simon et al., 2017, Wichert et al., 2013). Hupbach and colleagues have argued that such intrusions do not only reflect source memory failures, since they were generally unidirectional (i.e., later objects were reported as part of the activated-interfered list, but not vice versa; Hupbach et al., 2009). However, such unidirectionality cannot be assessed by counterbalanced interference, and may be an inseparable part of the experimental paradigm. Reconsolidation interference evidenced by retrieval failure outside the domain of intrusions remains quite sparse.

Although initial findings of behavioral reconsolidation interference generated excitement about theoretical and applicative implications of the phenomenon, there have been some recent reports of failures to find this effect. For example, Hardwicke and colleagues (Hardwicke and Shanks, 2016, Hardwicke et al., 2016) have reported that reconsolidation interference could not be demonstrated for procedural learning. In clinical applications of reconsolidation interference to the treatment of phobia (fear of flying), Maples-Keller et al. (2017) found no effect of the intervention on reported anxiety or behaviors, although they did observe effects on physiological measures of arousal. Jones, Pest, Vargas, Glisky, and Fellous (2015) failed to find effects of context-reinstatement potentiation of reconsolidation interference on object recall in older adults. Similarly, the studies herein reported failed to demonstrate reconsolidation interference to object picture recognition in a within-subject paradigm, despite numerous attempts to titrate reminder strength parameters. These findings, like those of Hardwicke and colleagues, do not necessarily represent evidence belying the phenomenon. However, these data underscore the evanescence of behavioral reconsolidation interference in human episodic memory, and provide some indications of what might be boundary conditions for its demonstration.
